# Relationship between pregnancy, delivery, and ileal pouch-anal anastomosis for inflammatory bowel disease: a retrospective chart review

**DOI:** 10.1093/crocol/otag048

**Published:** 2026-06-03

**Authors:** Sarah C House, Varun Srikanth, Parul Tandon, Katie O’Connor, Brenda O’Connor, Gajuna Mathiyalagan, Cynthia Maxwell, Erin Kennedy, John W Snelgrove, Anthony de Buck van Overstraeten, Mantaj Brar, Vivian Huang

**Affiliations:** Division of Gastroenterology and Hepatology, University of Toronto, Toronto, Ontario, Canada; Division of Gastroenterology, Mount Sinai Hospital, Toronto, Ontario, Canada; Division of Gastroenterology, Mount Sinai Hospital, Toronto, Ontario, Canada; Division of Gastroenterology and Hepatology, University of Toronto, Toronto, Ontario, Canada; Division of Gastroenterology and Hepatology, University Health Network, Toronto, Ontario, Canada; Institute of Health Policy, Management and Evaluation, University of Toronto, Toronto, Ontario, Canada; Division of Gastroenterology, Mount Sinai Hospital, Toronto, Ontario, Canada; Division of Gastroenterology, Mount Sinai Hospital, Toronto, Ontario, Canada; Division of Gastroenterology, Mount Sinai Hospital, Toronto, Ontario, Canada; Division of Gastroenterology and Hepatology, University of Toronto, Toronto, Ontario, Canada; Division of Gastroenterology, Mount Sinai Hospital, Toronto, Ontario, Canada; Women’s College Hospital, Toronto, Ontario, Canada; Division of Gastroenterology and Hepatology, University of Toronto, Toronto, Ontario, Canada; Division of Gastroenterology, Mount Sinai Hospital, Toronto, Ontario, Canada; Institute of Health Policy, Management and Evaluation, University of Toronto, Toronto, Ontario, Canada; Division of Gastroenterology and Hepatology, University of Toronto, Toronto, Ontario, Canada; Division of Gastroenterology, Mount Sinai Hospital, Toronto, Ontario, Canada; Institute of Health Policy, Management and Evaluation, University of Toronto, Toronto, Ontario, Canada; Women’s College Hospital, Toronto, Ontario, Canada; Division of Gastroenterology and Hepatology, University of Toronto, Toronto, Ontario, Canada; Division of Gastroenterology, Mount Sinai Hospital, Toronto, Ontario, Canada; Division of Gastroenterology and Hepatology, University of Toronto, Toronto, Ontario, Canada; Division of Gastroenterology, Mount Sinai Hospital, Toronto, Ontario, Canada; Division of Gastroenterology and Hepatology, University of Toronto, Toronto, Ontario, Canada; Division of Gastroenterology, Mount Sinai Hospital, Toronto, Ontario, Canada

**Keywords:** inflammatory bowel disease, ileal-pouch anal anastomosis, pregnancy, delivery

## Abstract

**Background:**

Pregnant women with inflammatory bowel disease (IBD) and an ileal pouch-anal anastomosis (IPAA) often receive conflicting medical advice regarding mode of delivery, and healthcare providers may have diverging opinions. Cesarean delivery is commonly performed out of concern of injuring the anal sphincter during vaginal delivery, causing poorer long-term pouch function. This study aims to describe rates of vaginal and cesarean delivery, complication rates and assess the impact of pregnancy and delivery on pouch function for patients with IBD and an IPAA.

**Methods:**

This retrospective single center chart review study included patients with IBD, an IPAA, and a completed pregnancy between January 1, 2002 and February 1, 2021. Patient demographics, IBD diagnosis, IPAA procedure details, mode of delivery and complications, and pouch function surrounding pregnancy were compared using descriptive statistics.

**Results:**

Sixty-two patients completed 85 pregnancies. Eighty-one percent of pregnancies had a cesarean delivery (69/85); of these, 51 (73.9%) were planned elective cesareans while 18 (26.1%) were urgent. Among the planned elective cesareans, 51.6% were indicated to prevent injury to the anal sphincter and preserve pelvic pouch function. Immediate risks of cesarean and vaginal delivery were similar to the general population. Rates of delivery intervention with episiotomy were high (50% of vaginal deliveries). All vaginal deliveries took place in the later 10 years of the study period. Pouch function postpartum was infrequently documented.

**Conclusions:**

Pregnant patients with IBD and an IPAA frequently had a cesarean delivery, often to avoid anal sphincter injury from vaginal delivery and preserve pelvic pouch function. Delivery practices shifted in the past 10 years to include vaginal delivery, likely reflecting an increased perceived safety of this delivery mode.

## Graphical Abstract

**Graphical Abstract:**
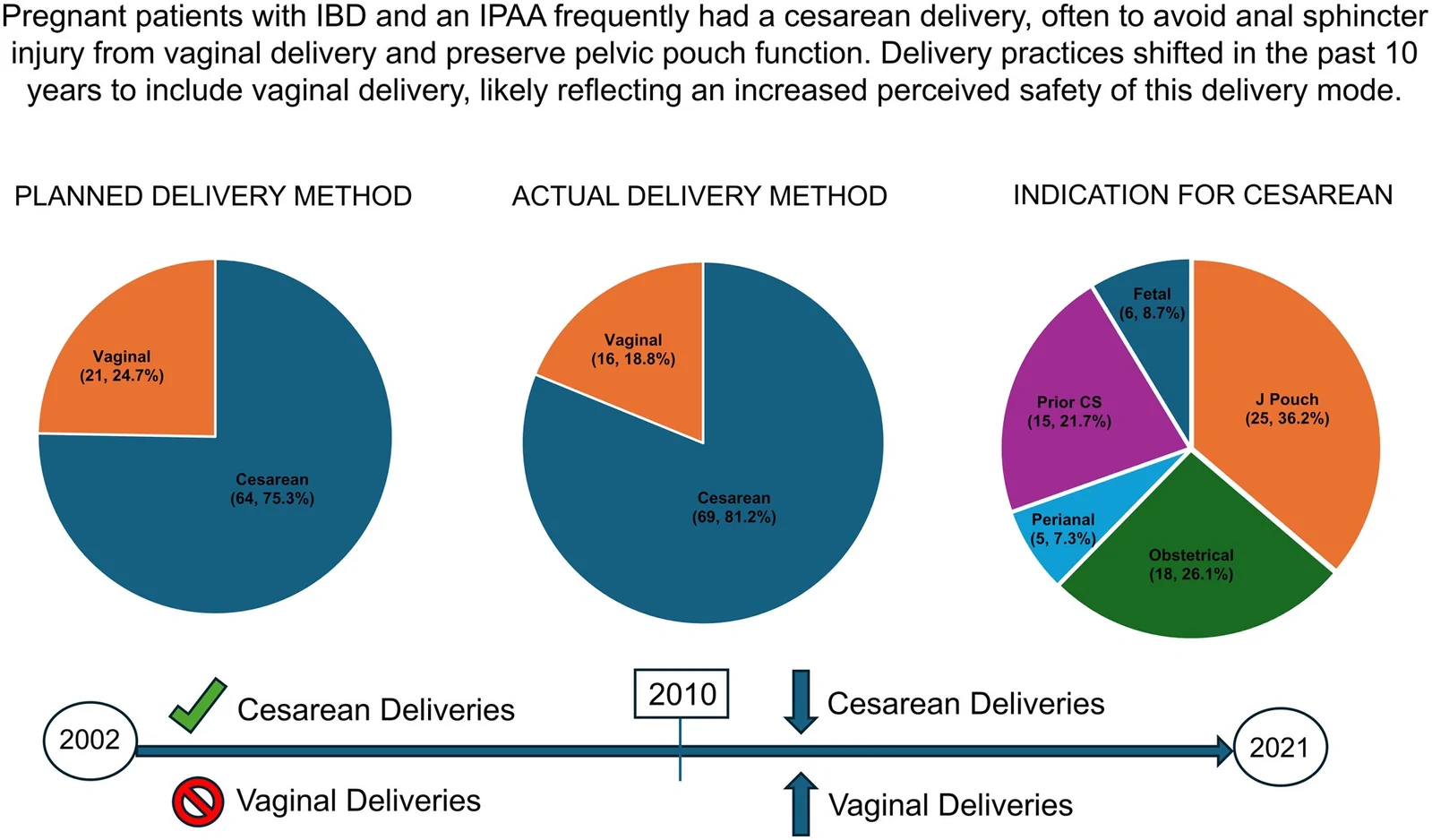

Key Messages

- **What is already known?** Pregnant patients with IBD and IPAA typically deliver via cesarean out of concern of damaging the anal sphincter during vaginal delivery causing long-term issues with pelvic pouch function.
- **What is new here?** Cesarean delivery rates to protect the pelvic pouch remain high. Use of vaginal delivery emerged in the past 10 years. The rate of episiotomy use is high.
- **How can this study help patient care?** The emergence of vaginal delivery in this population may reflect increased perceptions of safety in this mode of delivery. These results may encourage other institutions to analyze their own practice and reflect on whether their delivery recommendations are in line with the most recent evidence.

## Introduction

Ulcerative colitis (UC) is a type of inflammatory bowel disease (IBD) that is limited to the colon and rectum. Patients with UC would be recommended to have a proctocolectomy for several reasons which include medical refractory disease, colon cancer or dysplasia, or complications such as acute severe UC. An ileal pouch-anal anastomosis (IPAA), commonly referred to as a “pouch,” often “J-pouch,” is typically offered to allow patients to maintain continence.^1^ Most women with IBD will be diagnosed before or during their reproductive years,^2^^,^^3^ and thus a proportion of patients with IBD may have an IPAA at conception.

Pregnant patients with IPAA often receive conflicting delivery recommendations from clinicians.^4^ A study by Remzi et al.^5^ reported that 44% of their pregnant patients with IPAA were recommended to have a vaginal delivery by their obstetrician. However, only 8% received the same recommendation from their colorectal surgeon. Specialists recommending cesarean delivery do so based on reported risks of anal-sphincter damage and functional impairment from vaginal birth as compared to cesarean delivery.^4–7^ In contrast, many other studies have not reported any deterioration in pouch function from vaginal birth.^4^^,^^8–11^

Long-term effects of vaginal delivery and cesarean delivery on pouch function is unknown. Occult sphincter injury may not become clinically significant for many years.^6^ A recent Cochrane review^12^ investigating the impact of surgical therapies for IBD on female fertility and pregnancy outcomes concluded that high-quality data addressing this topic is lacking. This conclusion underlines the importance of continued investigation of this topic to inform clinical practice guidelines for pregnant patients with IPAA. As such, we aimed to evaluate the rates of vaginal and cesarean delivery as well as rates of delivery-related complications for patients with IBD who had an IPAA. The second aim is to determine the impact of pregnancy and delivery on pouch function in these patients.

## Materials and methods

This was a retrospective study at a quaternary care center that serves patients from across Ontario with medical complications such as IBD during pregnancy through its specialized care. This study was approved by the Research Ethics Board and was performed in accordance with the principles of the Declaration of Helsinki.

### Study population

Eligible patients were those who had a confirmed diagnosis of IBD, having had an IPAA procedure, and who delivered at our center between January 1, 2002 and February 1, 2021. To identify these patients, we collated data from multiple institutional databases, which included the high-risk obstetric clinic, (this institution) inpatient records—for diagnosis, pregnancy, and IPAA procedures—pouchoscopy records, and a surgery clinic database (see Supplementary Material 1 for details on patient identification approach).

These lists were manually screened for a diagnosis of IBD (UC or CD), history of IPAA surgery before conception of the pregnancy of interest, documentation of delivery, availability of delivery records for chart review. Patients were included if they had a diagnosis of IBD (UC or CD), had an IPAA prior to conception, and completed a pregnancy between January 1, 2002 and February 1, 2021.

### Outcomes

Variables related to patient demographics at the time of IBD diagnosis and at the time of conception (IBD diagnosis, age, prior deliveries) and medical history (IBD diagnosis at time of colectomy and details of the IPAA procedure), primary outcomes (delivery mode and complications), and secondary outcomes (pouch function during pregnancy and after delivery) were extracted from the hospital electronic medical records (includes electronic and scanned inpatient and outpatient notes) from the gastroenterology outpatient EMR (since August 2017, but it includes scanned paper files from pre-August 2017) and from the surgery clinic database. Regarding delivery mode, we documented the planned delivery mode (vaginal or cesarean section). We also documented the actual delivery mode (vaginal or cesarean section) and if elective or urgent cesarean section. Those who planned to have a scheduled cesarean section but ended up having an earlier delivery due to preterm labor or other cause (fetal distress) we classified as urgent. Regarding vaginal delivery, we reviewed the delivery records for episiotomy, presence and grade of perineal tear, and instrument assist. For cesarean deliveries, we documented the indication for elective/planned procedures (J-pouch, perianal disease, prior cesarean section, maternal indication [e.g., pre-eclampsia], obstetrical indication [e.g., placental position], fetal indication [e.g., breech], patient preference, physician-directed, or unknown). For urgent cesarean sections, the same categories were used, with the addition of failed vaginal delivery as an obstetrical indication. If there were multiple indications noted in the chart, we listed the primary indication based on what was specified in the obstetric reports. The term was defined by a gestational age ≥ 37 weeks at delivery; otherwise, “term” or “full term” stated in the delivery notes.

### Statistical analysis

Continuous data were plotted in a histogram to test for normal distribution. All data were considered non-normally distributed and are presented as medians and interquartile ranges (IQR). Categorical data are presented as frequency (*n)* and relative frequency (%). To test differences between medians, the Mann-Whitney U test was used. Chi-square or Fisher’s exact were used for categorical data. For sparse, > 2x2 tables, exact *P*-value were generated with a 10 000-sample Monte Carlo procedure. Effect sizes are reported as Cramer’s V. *P* < .05 was considered statistically significant. All statistics were calculated in SPSS Version 29.0.1.0 (SPSS, Chicago, Illinois, USA).

## Results

### Patient characteristics

A total of 1113 patient charts were identified from the lists described in the Methods section, and after screening, 62 patients met inclusion criteria (Table 1). Of the 62 patients, most had UC and were diagnosed at a median age of 18 years (IQR 14.8-23.0). Patients underwent IPAA surgery at a median age of 24 years (IQR 19.0-27.0), and most were completed via laparotomy rather than through a laparoscopic approach. The indication for IPAA (stage 1 colectomy) was noted for 43 patients, with the primary reason being medically refractory disease. The median interval between IPAA (stage 2 pouch creation) and conception was 9 years (IQR 6-14).

**Table 1:** Patient characteristics.

	Total patients included (*n* = 62)
**IBD diagnosis at initial diagnosis:**	
**UC, *n* (%)**	58 (93.6)
**CD, *n* (%)**	4 (6.5)
**Age at diagnosis, median, years (IQR)** ^a^	17.0 (15.0-23.0)
**Age at IPAA, median, years (IQR)** ^b^	24.0 (18.0-27.0)
**IPAA procedure technique** ^c^	
**laparotomy, *n* (%)**	54 (87.1)
**laparoscopy, *n* (%)**	5 (8.1)
**IPAA stages** ^d^	
**one stage, *n* (%)**	1 (1.6)
**two stages, *n* (%)**	42 (67.7)
**three stages, *n* (%)**	16 (25.8)
**Indication of colectomy** ^d^	
**Medically refractory**	51 (82.3)
**Disease related complication**	3 (4.8)
**Other**	1 (1.6)
**Unknown**	7 (11.3)
**Completed pregnancies, *n***	85
**IBD diagnosis at conception**	
**UC, *n* (%)**	78 (91.8)
**CD, *n* (%)**	7 (8.2)
**Twin pregnancies, *n***	5
**Age at conception for first pregnancy, median years (IQR)** ^e^	31.0 (29.0-35.0)
**Maternal age at delivery, median years (IQR)**	33.7 (31.4-36.9)

Abbreviations: CD, Crohn’s disease; IBD, inflammatory bowel disease; IPAA, ileal pouch–anal anastomosis; IQR, interquartile range; UC, ulcerative colitis.

a Missing information *n* = 1.

b Missing information *n* = 1.

c Missing information *n* = 3.

d Missing records *n* = 3.

e Missing information *n* = 3.

**Table 2:** Vaginal delivery outcomes.

	**Total vaginal deliveries (*n* = 16**)
**Assisted, *n* (%)^a^**	5 (31)
**Vacuum, *n* (%)**	3 (19)
**Forceps, *n* (%)**	2 (13)
**Episiotomy, *n* (%)^b^**	8 (50)
**Assisted, *n* (%)**	4 (25)
**Tears, *n* (%)^c^**	8 (50)
**First degree, *n* (%)**	3 (19)
**Second degree, *n* (%)**	3 (19)
**Third degree, *n* (%)**	2 (13)
**Fourth degree, *n* (%)**	0 (0)
**Nulliparous, *n* (%)**	5 (31)
**No tears or episiotomy, *n* (%)**	1 (6)

a Missing information *n* = 3.

b Missing information *n* = 1.

c Missing information *n* = 2.

Across all patients, 85 pregnancies were completed; 23 patients had a second pregnancy during the time frame. The median maternal age at first conception was 31.0 years (IQR 29.0-35.0). Median age at delivery was 33.7 years (IQR 31.4-36.9). There were 5 twin pregnancies. More than half of the pregnancies in our dataset (55.3%) were nulliparous.

### Delivery mode trends over time

**Figure 1:**
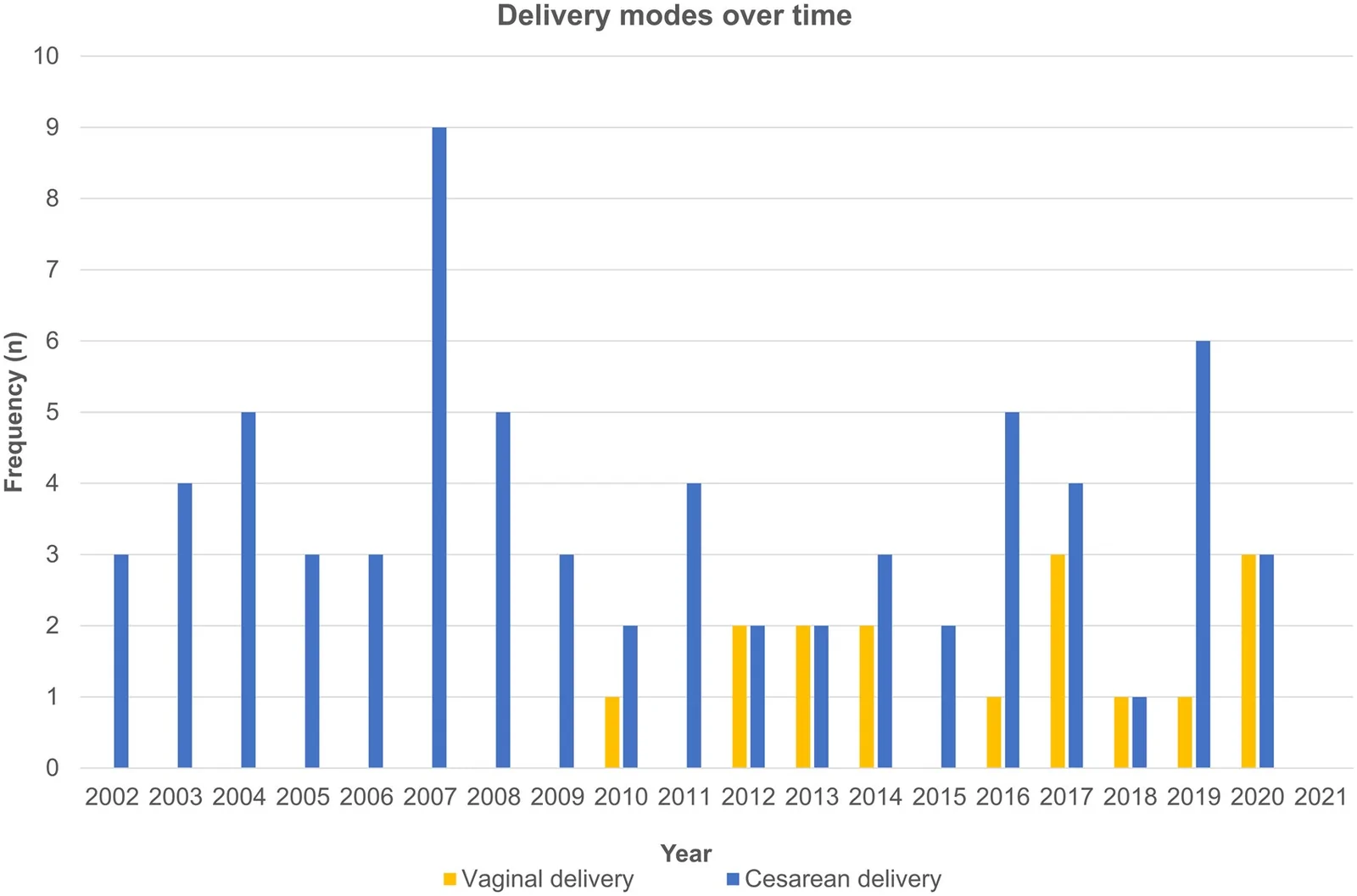
Delivery modes over time.

There were 69 (81.2%) cesarean deliveries and 16 (18.8%) vaginal deliveries. As shown in Figure 1, there were no vaginal deliveries between 2002 and 2009, whereas from 2010 to 2021 the cesarean rate decreased by 32% (*P* < .001; Cramer’s V = 0.403).

There was a trend toward a longer duration of time between IPAA and conception in vaginal deliveries (10 years [IQR 9.0-22.0]), but this was not statistically significant compared to cesarean deliveries (8.5 years [IQR 6.0-13.0]; *P* = .403).

### Pregnancy outcomes

The gestational age at delivery was missing for 6 pregnancies. For 3 of them, term-at-delivery was determined from the delivery notes, resulting in confirmed term or preterm status for 82 patients. Sixty-seven pregnancies (78.8%) resulted in a delivery at term; the preterm delivery rate was 21.0%. The median gestational age at birth was 38.4 weeks (IQR 37.1-39.0).

Data on gestational complications were available for 82 patients (3 missing). Gestational complications included gestational diabetes, intrahepatic cholestasis of pregnancy, placenta previa, placental insufficiency, and short cervix. Ten patients (12.2%) had at least one gestational complication, and some experienced more than one. Seven occurred among cesarean deliveries (10.1%) and 3 among vaginal deliveries (18.8%). Among the cesarean deliveries, complications were documented in 16.7% of urgent cases compared with 7.8% of elective cases (*P* = .411; Cramer’s V = 0.160).

Infants delivered through cesarean were born at a median gestational age of 38.0 weeks (IQR 37.3-39.0), compared with 39.1 weeks (IQR 37.1-39.7) for vaginal deliveries (*P* = .058). Singleton pregnancies had a longer median gestation (38.4 weeks [IQR 37.3-39.0]) than twin pregnancies (36.6 weeks [IQR 33.4-36.9]; *P* = .007). The median birthweight of babies delivered via cesarean was 3.09 kg (IQR 2.72-3.57) compared with 3.32 kg (IQR 3.00-3.45) for vaginal deliveries (*P* = .695). Median birthweights were 2.70 kg (IQR 2.23-2.90) among twins and 3.22 kg (IQR 2.85-3.66) among singletons (*P* = .052).

#### Cesarean delivery outcomes

**Figure 2:**
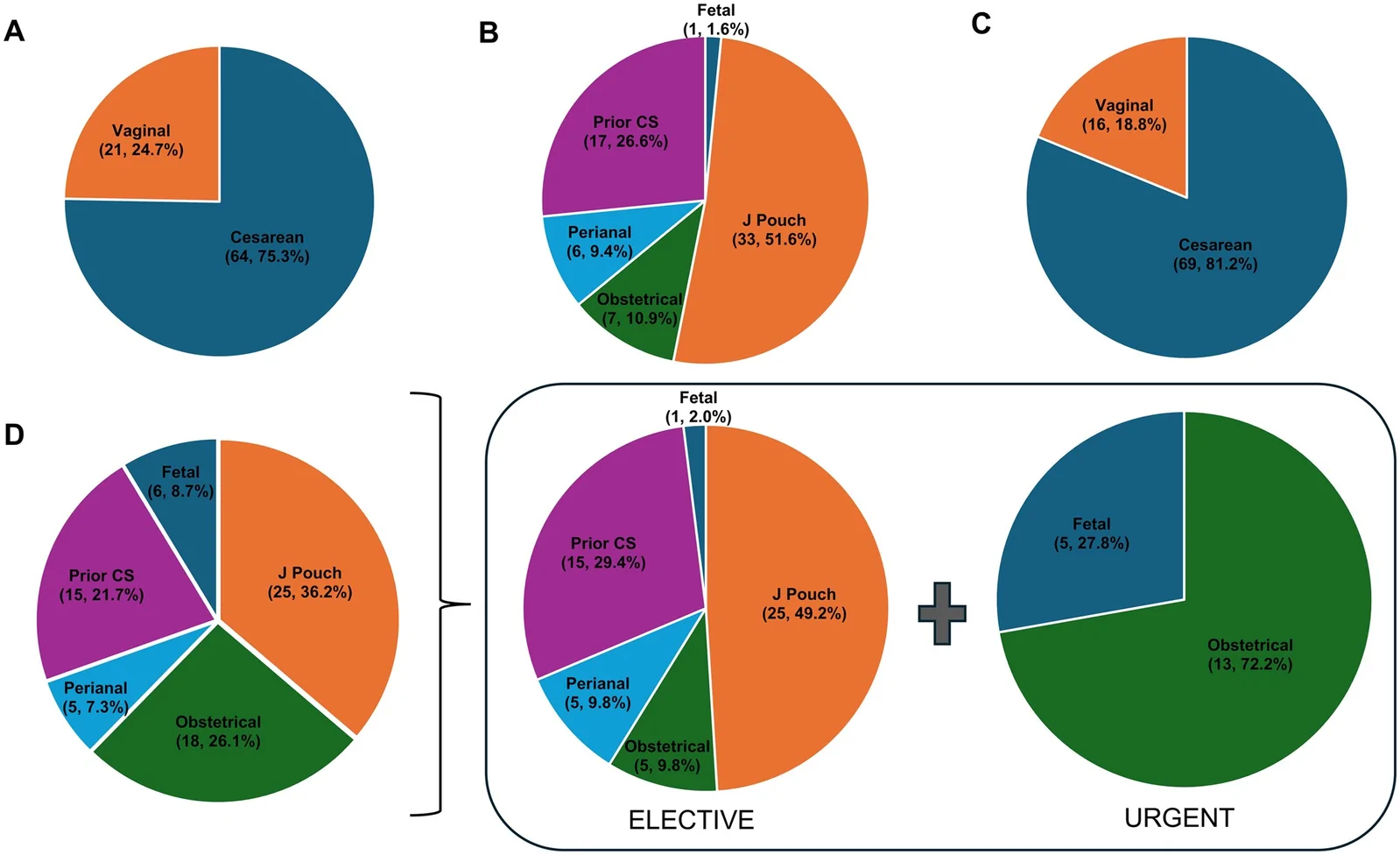
Delivery outcomes and cesarean delivery indication (A) Planned delivery method, (B) Indication for elective cesarean delivery, (C) Actual delivery method, and (D) indication for cesarean delivery. CS, cesarean section.

Of the 85 pregnancies, 64 (75.3%) were planned for cesarean section and 21 (24.7%) for vaginal delivery (Figure 2A). The most common primary indications for a planned elective cesarean were J-pouch protection (51.6%), prior cesarean section (26.6%), and perianal disease (9.4%), corresponding to 33, 17, and 6 cases, respectively (Figure 2B).

Five patients underwent conversion from a planned vaginal delivery to an urgent cesarean, resulting in 69 actual cesarean deliveries (Figure 2C). Of these, 51 (73.9%) were elective and 18 (26.1%) were urgent. The most common primary indications for actual cesarean deliveries were J-pouch protection (25, 36.2%), obstetrical indications (18, 26.1%), and prior cesarean section (15, 21.7%) (Figure 2D).

Among the 18 urgent cesarean sections, 13 were converted from elective/planned cesarean delivery and 5 from planned vaginal delivery (arrest of dilatation in four cases and fetal malpresentation in one). The main reasons for conversion to urgent cesarean delivery were obstetrical (13, 72.2%—spontaneous rupture of membrane or labor, preterm rupture of membrane or labor, preeclampsia, and sepsis) and fetal (5, 27.8%) (Figure 2D). All 5 conversions from vaginal to cesarean delivery occurred in nulliparous patients, and 4 occurred before 2010.

Among the 23 patients with two or more pregnancies, 18 (78.0%) planned repeat cesarean delivery and 5 (22.0%) planned vaginal delivery. One of the latter required urgent conversion, yielding a total of 19 cesarean deliveries in this subgroup.

There were 47 nulliparous pregnancies in total, of which 40 (85.1%) were delivered by cesarean section compared with 28 of 36 multiparous pregnancies (77.8%). Among the 40 nulliparous cesarean deliveries, 4 (10.0%) were performed due to active perianal disease, 8 (20.0%) for obstetrical reasons, 6 (15.0%) for fetal reasons, and 22 (55.0%) for pelvic pouch protection.

Of the 69 cesarean deliveries, 18 were urgent and 51 were elective; out of all of them, 56 (81.2%) were uncomplicated. Twelve deliveries (17.4%) were associated with intrapartum complications, including fetal distress, vacuum assistance, urgent conversion, placental abruption, breech presentation, fetal loss, and one case of fetal intubation post-delivery. One was missing details about complications. Additionally, 3 deliveries (4.3%) involved intraoperative complications, including dense adhesions requiring an extraperitoneal approach, significant intraoperative hemorrhage managed with a Bakri balloon, and an incidental cystotomy repaired intraoperatively. Maternal and fetal complications during delivery occurred in 16.7% of urgent cesareans (3/18) and 7.8% of elective cesareans (4/51). Neonatal complications occurred in 4 cesarean deliveries: 27.3% of urgent (3/11) and 1.7% of elective (1/58).

Forty-seven cesarean deliveries (68.0%) had no documented post-operative complications. The remaining 22 cesarean deliveries (32.0%) were associated with post-operative complications. Bowel obstruction occurred in 5 cases, and ileus and anemia were each reported in 3 cases. Pouchitis, hemorrhage, increased stool frequency, abdominal pain, and nausea were each documented in 2 cases. Single cases included volvulus requiring urgent laparotomy, fistula, pelvic pain, difficulty with evacuation, wound infection, bacteremia, atelectasis, wound dehiscence, pouch ulceration, and gastritis within a year of cesarean delivery.

Four neonatal complications (5.8%) were documented among the cesarean deliveries: 3 of 11 (27.3%) urgent cases and 1 of 58 (1.7%) elective cases (*P* = .012; Monte Carlo 99% CI, 0.010-0.015; Cramer’s V = 0.402).

In 6 pregnancies, the maternal diagnosis changed from UC at initial diagnosis to Crohn’s disease at conception. All 6 planned cesarean delivery—indications were 1 perianal, 3 for J-pouch protection, and 2 for prior cesarean—and all 6 delivered by cesarean section—4 elective and 2 urgent following spontaneous labor.

#### Vaginal delivery outcomes

Of the 85 completed pregnancies, 16 deliveries were vaginal, representing 18.8% of the cohort (Table 2). Approximately a third of the deliveries (31%) were assisted by either obstetrical forceps or vacuum. Half of vaginal deliveries required episiotomy, including 80% of assisted deliveries and 36% of non-assisted deliveries. Perianal tears occurred in half of vaginal deliveries; only two cases involved a third-degree tear, and none involved a fourth-degree tear. Most of the patients who developed a tear were nulliparous.

Most vaginal deliveries (69.0%) were uncomplicated. Five patients experienced maternal complications. Nephrolithiasis and pouchitis each occurred in two patients. Single cases included incontinence, weakening of the anal sphincter, fecal leakage, acute renal injury, hypertension, inability to void, and sphincter and pudendal nerve injury. Two of the five patients experienced more than one complication: one had pouchitis with nephrolithiasis, and another had incontinence with inability to void.

## Discussion

We demonstrate high rates of cesarean delivery (81.2%) among pregnant patients seen at (this institution) with IBD and IPAA between 2002 and 2021. This cesarean delivery rate is much higher than in the general population in Canada (29.9% in 2019)^13^ and higher than what has been reported in previous studies of pregnant patients with IPAA. We observed that the original indication for the elective cesarean deliveries was to protect the IPAA (51.6%) and that the most common indication for conversion from elective to urgent cesarean was obstetrical, and conversion from vaginal delivery to urgent cesarean was arrest of dilatation. A 2017 systematic review^6^ reported a 56% rate of cesarean delivery (range 25%-76%, 44% were indicated for IPAA reasons) and a similar study^8^ including patients prior to 2002 reported a cesarean delivery rate of 49%.

In our study cohort, patients conceived their first child slightly older (*P* < .001) than the median age of mothers at first birth in Canada (median age 31 compared to 29.4 years in Canada)^14^ suggesting IBD diagnosis and IPAA surgery may delay childbearing. However, this comparison is limited, as national statistics include women without IBD and therefore do not isolate the effect of IPAA surgery. IPAA has been associated with reduced fertility and increased infertility in prior studies, which may contribute to differences in reproductive timing. Additionally, population-based studies show that women with IBD overall have altered reproductive patterns and lower pregnancy rates compared with women without IBD, and prior surgery further increases time to pregnancy.^15^^,^^16^ Therefore, the older age at conception observed in our cohort may reflect underlying IBD- and surgery-related reproductive factors rather than the effect of IPAA alone.

Most patients in our cohort delivered their babies at term; there was a 21.0% preterm rate. Conversely, 7.9% of live births in the general population of Canada are delivered preterm.^17^ This is also in contrast to reports in other studies suggesting IBD and IPAA have no impact on length of pregnancy.^18^ This difference may be due to the study’s small sample size but may also reflect a more medically complex population seen at (this institution) as a quaternary center. Patients in this cohort may also have a relatively higher IBD disease burden or complex obstetric history that may impact length of pregnancy.

The reason for the high rate of cesarean delivery in this cohort is unknown but may reflect the complexity of the patient presentation as well as provider recommendations and patient preferences. Additionally, previous research shows that colorectal surgeons are more likely to recommend cesarean delivery^19^ based on studies suggesting vaginal delivery may compromise IPAA function in the long-term. Access to this opinion may be higher when receiving care in a quaternary center as compared to a community center. The cesarean rate among nulliparous patients (85.1%) was comparable to the overall rate, indicating the overall rate is not impacted by a prior history of cesarean delivery. Interestingly, in this 20-year cohort, all the vaginal deliveries were in the later half between 2010 and 2021 (Figure 1), which may reflect an increased perceived safety of vaginal deliveries in this population.

One of the most severe complications of vaginal delivery is third- and fourth-degree perineal tears, as they involve the anal sphincter and increase the risk of fecal incontinence.^20^ Rates of high-degree tears during vaginal delivery in this study were relatively low (*n* = 2 [13%]) and were similar to general rates in Canada (3.1-16.4 per 100 deliveries)^21^. Episiotomy was used in 50% of vaginal deliveries in this cohort, 80% of operative deliveries and 36% of non-operative deliveries. This rate is much higher than that of the general population (43.2% of operative vaginal deliveries and 6.5% of spontaneous vaginal deliveries)^22^ likely reflecting efforts to protect the perineum during delivery. Episiotomy has been shown to have protective effects against third- and fourth-degree tears,^22^ and given the severe consequences of anal sphincter injury, obstetricians likely to have a lower threshold to perform an episiotomy in this patient population than in patients without a pouch.

Cesarean delivery exposes a patient to intraoperative complications that are not present during vaginal deliveries. For patients with a history of abdominal surgeries, as is true for those with an IPAA, the risk of damage to neighboring organs such as bowel and bladder during cesarean delivery is thought to be higher due to adhesions causing distortion of normal anatomy.^20^ Despite this, only 4.3% of cesarean deliveries in our study experienced intraoperative complications. This is mildly elevated compared to rates in the general population of 2.7% of women with planned cesarean delivery experiencing severe morbidity,^20^ suggesting this population may not be at increased risk of intraoperative complications. In our cohort, gestational complications occurred in 10.1% of cesareans, delivery-related complications in 17.4% (comprising 16.7% of urgent and 7.8% of elective cesarean deliveries), and neonatal complications in 5.8%, mirroring the higher morbidity seen when labor necessitates urgent conversion.

The risks of vaginal and cesarean delivery among the general population are well known with the risk of severe morbidity being much higher from planned cesarean (2.7%) compared to vaginal delivery (0.9%).^20^ In the context of patients with IBD and IPAA, there is an added concern of damaging the pelvic pouch after having endured several surgeries preceded by years of severe symptomatic disease that may elevate the perceived risk of vaginal delivery. High national rates of cesarean delivery may also contribute to patients’ perceived safety of this mode of delivery and impact their preference toward its use.^20^ However, this leads us to wonder whether patients are being overexposed to known severe risks of cesarean delivery due to concerns of risks of vaginal delivery that are inconclusively substantiated in the literature.^4^ Even among the general population, there is mixed evidence surrounding whether cesarean delivery has a protective role^23–27^ as recent research suggests pregnancy itself may increase the risk of developing anal incontinence.^28^

Regarding possible reasons for the shift toward vaginal delivery over the years, we believe that this likely represent a true shift in counseling around options for delivery mode. At our center, a multidisciplinary clinic commenced around 2010, combining maternal fetal medicine, obstetrics, gastroenterology, and colorectal surgery teams. These teams provide input during pregnancy care and with delivery planning. There is now more discussion around mode of delivery options between providers amongst each other and between providers and patients. The Toronto Consensus Statements for the management of IBD in pregnancy were published in 2016, but the preparation for this guideline commenced around 2014.^29^ Clinical work started around 2010/2011 and so likely the changes in rates of vaginal birth are related to this as well. Increased patient engagement and education regarding delivery mode options may also have contributed. Clinical recognition of interventions related to vaginal delivery that lower the risk of OASIS (e.g. episiotomy) may have further influenced counseling practices.

The cesarean section rate was still high in this cohort. There may be secular trends over time for increased cesarean section rates in the general population (e.g. older maternal age at first pregnancy). The high-risk population at our single center may have contributed to more patients having additional indications for cesarean delivery. Many patients still choose cesarean delivery after counseling about delivery options. Also, IPAA is still a relative indication for cesarean section in Toronto^29^ and international guidelines such as the American Gastroenterological Association guidelines published in 2019^2^.

### Limitations

There are a few limitations to this study. This was a single-center retrospective study, and thus the findings may not be the same in other centers. This study was designed as a historical case series of pregnant patients with IPAA and was not intended as a comparative cohort study. A control group of pregnant patients with IBD without IPAA was not included, as systematic data collection for non-surgical IBD pregnancies was not established at this institution during much of the study period. Additionally, in a center providing specialized care, many patients travel to seek obstetrical care during their pregnancy and then return to their local physician. As a result, notes describing any maternal comorbidities, pouch function surrounding pregnancy, as well as post-delivery complications were largely absent from patient charts, which was a major limitation to investigating these outcomes. The availability of complete records was also lacking for earlier years because of the way the hospital and outpatient clinic notes were stored. Objective assessments—pouchoscopy, anal manometry, or validated clinical scoring—were either unavailable or inconsistently available in postpartum notes to meaningfully report on pouch function before and after delivery; hence, this limited our efforts to address the second objective of this study.

## Conclusion and future directions

This study has revealed the high rates of cesarean delivery (81%) among patients at [this institution] with IBD and IPAA over 2 decades as well as uncovering the tendency for patients to have a cesarean delivery to protect their IPAA. Immediate risks of cesarean and vaginal delivery were similar to the general population. Episiotomy was utilized at high rates, which was most likely implemented to protect against anal sphincter injury. Vaginal delivery was only utilized after 2009, potentially reflecting increased perceived safety of this mode of delivery in the latter half of the study period. While the impact of pregnancy and delivery on pouch function requires further investigation, the aforementioned findings offer valuable practice-level insights. With this in mind, no large, long-span prospective studies have assessed modes of delivery for patients with IBD and an IPAA, which is important as functional decline due to occult anal sphincter injury commonly occurs in the fifth and sixth decades of life.^30^ Long-term impacts of cesarean and vaginal delivery remain unknown.

This research field has been stagnated by the sparse evidence and lack of prospective studies investigating the long-term impact of cesarean versus vaginal delivery on pouch function. Retrospective chart reviews are limited by the data available within patient charts. Prospective multi-center studies investigating this topic using validated symptom scoring scales and anal manometry are sorely needed to shed light on the true risks of delivery modes. It is certainly this lack of quality evidence that leads to the diversity of opinions around the mode of delivery among obstetricians, gastroenterologists, and colorectal surgeons.

From a clinical perspective, there is a great need for collaboration among specialists to unify patient advice. This collaboration can be realized on the patient level by increased communication between specialties prior to providing delivery advice to a patient. Lastly, we encourage institutions who deliver patients with IBD and IPAAs to study their delivery practices and reflect on whether they are in line with the most recent evidence.

## Supplementary material

Supplementary material is available at *Crohn’s and Colitis 360* online.

## Supplementary Material

otag048_Supplementary_Data

## Data Availability

Data not publicly available but can be provided upon request.
