# Supplementary material for: Relationship between pregnancy, delivery, and ileal pouch-anal anastomosis for inflammatory bowel disease: a retrospective chart review
Source: Crohns Colitis 360. 2026 Jun 3;8(3):otag048. doi: 10.1093/crocol/otag048 (PMC13348844; doi:10.1093/crocol/otag048)
Supplement: otag048_Supplementary_Data [file otag048_supplementary_data.docx]

Supplementary Material

(1) Patient Identification Approach

1. High risk obstetrics clinic: A list of patients identified as having Crohn’s disease or Ulcerative Colitis from the centre’s high risk obstetrics clinic database of patients seen between January 1, 2002 to February 1, 2021.
2. Hospital Records for IBD and pregnancy: A list of patients seen at our centre between January 1, 2002 to February 1, 2021 who were identified with 1) International Classification of Diseases 9^th^ revision (ICD-9) and 10^th^ revision (ICD-10) codes for IBD and pregnancy AND 2) O00.0-O99.809 (pregnancy and pregnancy-induced complications).
3. Hospital Records for IPAA: A list of patients who had undergone an IPAA procedure at our centre labelled with any of the following ICD-9 and ICD-10 CCI codes: 1.NK.84.RR-XX-G (Construction or reconstruction, small intestine using ileum (to construct ileostomy pouch)), 1.NQ.84.DA-XX-G (Construction or reconstruction, rectum using endoscopic [laparoscopic, laparoscopic-assisted, hand-assisted] approach with ileum (to construct pouch)), 1.NQ.84.LA-XX-G (Construction or reconstruction, rectum using open approach with ileum (to construct pouch)), 1.NQ.89.SF-XX-G (Excision total, rectum abdominal [anterior] approach pouch formation), 1.NQ.89.KZ-XX-G (Excision total, rectum abdominoperineal approach pouch formation), 4863 (ICD9) (Other anterior resection rectum)
4. Hospital Records for female patients who had a pouchoscopy: A list of female patients who had undergone a pouchoscopy procedure at our centre using the following criteria: 2OW70BA, 2OW70BN, 0121 (ICD9).
5. Surgery clinic database for female patients with IPAA: A list of female patients who had undergone an IPAA procedure at our centre was extracted from the centre’s surgery clinic database.
